# Human Virus Genomes Are Enriched in Conserved Adenine/Thymine/Uracil Multiple Tracts That Pause Polymerase Progression

**DOI:** 10.3389/fmicb.2022.915069

**Published:** 2022-06-01

**Authors:** Emanuela Ruggiero, Enrico Lavezzo, Marco Grazioli, Irene Zanin, Maja Marušič, Janez Plavec, Sara N. Richter, Stefano Toppo

**Affiliations:** ^1^Department of Molecular Medicine, University of Padua, Padua, Italy; ^2^Slovenian NMR Centre, National Institute of Chemistry, Ljubljana, Slovenia; ^3^CRIBI Biotechnology Center, University of Padua, Padua, Italy

**Keywords:** viruses, conservation, adenines, non-canonical structures, polymerase progression

## Abstract

The DNA secondary structures that deviate from the classic Watson and Crick base pairing are increasingly being reported to form transiently in the cell and regulate specific cellular mechanisms. Human viruses are cell parasites that have evolved mechanisms shared with the host cell to support their own replication and spreading. Contrary to human host cells, viruses display a diverse array of nucleic acid types, which include DNA or RNA in single-stranded or double-stranded conformations. This heterogeneity improves the possible occurrence of non-canonical nucleic acid structures. We have previously shown that human virus genomes are enriched in G-rich sequences that fold in four-stranded nucleic acid secondary structures, the G-quadruplexes.Here, by extensive bioinformatics analysis on all available genomes, we showed that human viruses are enriched in highly conserved multiple A (and T or U) tracts, with such an array that they could in principle form quadruplex structures. By circular dichroism, NMR, and *Taq* polymerase stop assays, we proved that, while A/T/U-quadruplexes do not form, these tracts still display biological significance, as they invariably trigger polymerase pausing within two bases from the A/T/U tract. “A” bases display the strongest effect. Most of the identified A-tracts are in the coding strand, both at the DNA and RNA levels, suggesting their possible relevance during viral translation. This study expands on the presence and mechanism of nucleic acid secondary structures in human viruses and provides a new direction for antiviral research.

## Introduction

The genetic information of organisms is stored into deoxyribonucleic acid (DNA), which is generally arranged in a supercoiled form, further stabilized by ancillary proteins. The typical double-stranded DNA conformation is the B-DNA form, but in certain sequence and environmental conditions, the length and width of the double helix may vary, giving rise to different arrangements (Ghosh and Bansal, 2003; Richter et al., 2007). The duplex DNA can then be subjected to unnatural bending in case of specific sequence features or DNA-binding proteins (Haran and Mohanty, 2009; Drozdetski et al., 2019). During key cellular processes, the DNA unwinds to give a single-stranded form that may adopt multiple three-dimensional conformations, different from the canonical Watson and Crick double helix, such as bulges, hairpins, cruciform, triplex, quadruplex, and i-motif structures (Kaushik et al., 2016). This structural polymorphism relies both on the DNA primary sequence, and on the cellular environment (solution, hydration, salts), the presence of proteins or exogenous molecules, and superhelical stress (Kaushik et al., 2016). Unconventional DNA structures are normally formed transiently and promote (or prevent) certain cellular mechanisms, thus being strictly involved in the life cycle of cell. In some pathological conditions, such as amplification of a repeat region, they may also lead to the onset of a range of pathologies (Kaushik et al., 2016). Non-canonical nucleic acid secondary structures are being investigated as pharmacological targets in the treatment of multiple human diseases, such as cancer, neurodegenerative disorders, and infectious diseases (Tateishi-Karimata and Sugimoto, 2020). Indeed, alternative structures have been identified in the genome of several microorganisms (Saranathan and Vivekanandan, 2019), including viruses (Ruggiero and Richter, 2020).

Viruses parasite the eukaryotic or procaryotic cell to allow their own replication, adding a high variety to the types of nucleic acids that store the genetic information: besides double-stranded DNA, virus nucleic acids can also present DNA or RNA in several conformations, such as single-stranded, double-stranded, and fragmented (Modrow et al., 2013). This heterogenicity obviously further improves the possible occurrence of non-canonical nucleic acid structures.

We have previously shown that virus genomes are enriched in G-rich sequences that can fold in the non-canonical structures G-quadruplexes (G4s) (Lavezzo et al., 2018). G4s are formed by G-tetrads stabilized by Hoogsteen H-bonds (Supplementary Figure 1A). Two or more G-tetrads self-stack to form the G4, which is stabilized by electrostatic interactions with monovalent cations (Huppert, 2008). G4s are located in pivotal genomic regions, both in DNA and RNA, where their formation regulates several cellular processes, such as replication, transcription, translation, and recombination both in eukaryotic cells and viruses (Spiegel et al., 2019; Frasson et al., 2021; Lago et al., 2021). In addition to Gs, tetrads can also comprise other nucleobases (Kocman and Plavec, 2017) or be exclusively composed of adenine (A), cytosine (C), thymine (T), or uracil residues (U). Examples include the Hotoda’s sequence aptamer, which arranges into a G4 structure composed by one A-tetrad and four G-tetrads (Romanucci et al., 2019); the telomere repeats of *Saccharomyces cerevisiae*, a G4 made by both G and T-tetrads (Patel and Hosur, 1999); the human telomere RNA segment GUUAGGGU arranged into a complex G4, including four G, one A, and two U-tetrads (Xiao et al., 2017); a GCGC-tetrad that can form through both Hoogsteen and Watson and Crick H-bonds (Viladoms et al., 2009; Supplementary Figure 1).

We here provided evidence that virus genomes present conserved multiple A (and T or U) tracts that could in principle form quadruplex structures. We proved that this is not the case, but these tracts are biologically active, as they trigger pausing of polymerase.

## Results

### Detection of A and T Multiple Tracts in the Genome of All Human Viruses

While analyzing the genome of all known human viruses for the presence of conserved putative G4-forming tracts (Lavezzo et al., 2018), we observed a general conserved enrichment of A/T-tracts. A-tetrads in a G4 context have been reported by X-ray crystallography and NMR spectroscopy analysis to form Hoogsteen-type H-bonds between N(6)H_2_ that acts as a donor and N(1), N(3),and N(7) that act as acceptors, and in principle, an additional structure can be formed in the presence of the rare A tautomeric form, in which eight H-bonds can occur, analogously to G-tetrads (Supplementary Figures1A,B; Van Der Wijst et al., 2010). T and U-tetrads are stable in solution, and are characterized by H-bonds between O(4) and N(3)H residues (Supplementary Figures1C,D; Gu and Leszczynski, 2001). For all these structures, cations can act as conformational switches, modulating the association of nucleobases and the consequent geometry of the adopted structure (Haran and Mohanty, 2009). Prompted by this evidence, we scanned the genome of all human viruses for the presence of A quadruplex-like sequences (A-QLSs) with the tool QPARSE (Berselli et al., 2020), to detect tracts that could form quadruplex motifs, according to the same rules described for G4s. Similar to the previous search for putative G4 sequences (Lavezzo et al., 2018), we searched for tracts made of two, three, or four consecutive As as follows: [A(2)N(1–7)](3)A(2), [A(3)N(1–12)](3)A(3), and [A(4)N(1–12)](3)A(4), where “N” indicates any of the four nucleotides. The presence of A-QLSs in the antisense strand of viral genomes was assessed searching for T/U in the sense strand, to take into account the heterogeneity of the viral genomes (double-stranded vs. single-stranded genomes, DNA vs. RNA) and the different stages of viral genome replication and/or transcription, as the antisense strand corresponds to viral transcripts in single-stranded negative polarity RNA viruses [ssRNA(−)] and serves as a template for replication in single-stranded positive-polarity RNA viruses [ssRNA(+)].

**FIGURE 1:**
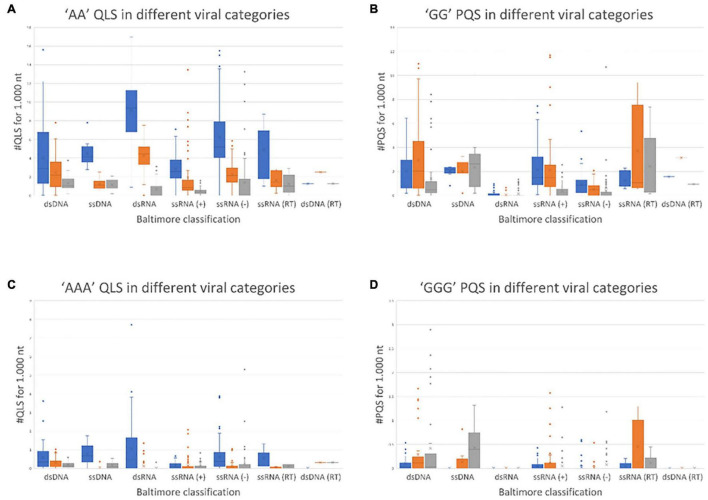
Normalized abundance of A-QLSs **(A,C)** and G-PQSs **(B,D)** in the genome of all human viruses, grouped by Baltimore class. Each panel refers to the specified type of island (AA and AAA for A-QLSs, GG and GGG for G-PQSs); boxplots are delimited by the first and third quartile and the inside horizontal line is the median value of the QLS distribution. Whiskers delimit all the points that fall above/below the third/first quartile ± 1.5 times of the interquartile range (IQR). Bar colors indicate the strand where the predicted patterns are found with respect to coding sequences and refer to concordant (same strand of a CDS, blue), discordant (opposite strand of a CDS, orange), and non-coding (no CDS overlaps that pattern, gray), respectively.

The results of this comprehensive search are shown in Figure 1, where viruses are grouped according to their Baltimore class and the amount of A-QLSs is normalized per 1,000 nucleotides. A-QLSs are classified, according to the strand they are found in, as “concordant” if they occupy the same strand of a coding sequence (CDS), “discordant” if they are in the complementary strand of a CDS, or “non-coding” if they are in a genomic region lacking CDS in both strands. A-QLSs with islands of length of two and three As (AA and AAA) were present in all Baltimore classes, but with clear differences. First, except for double-stranded DNA viruses (classes I and VII), concordant A-QLSs were more abundant than discordant or non-coding ones (Figure 1A), and this held true also for viruses belonging to class V, characterized by a single-stranded RNA genome with negative polarity. These data indicate that A-QLSs are preferentially present in the coding strand, and thus also at the viral mRNA level. Two A-QLSs were the exceptions: the Baltimore class I, which features viruses with double-stranded DNA genomes, and the Baltimore class VII, the only member of which is hepatitis B virus (HBV). The unbalanced presence of A-QLSs with respect to the coding strand is peculiar and not detected in G-putative quadruplex forming sequences (PQSs), which are evenly distributed in CDS and both strands among all Baltimore classes (Figure 1B).

A quadruplex-like sequences and G-putative quadruplex forming sequences with islands of length four were rarely present across all Baltimore classes (Supplementary Figure 2). When comparing the overall abundance of predicted A-QLSs and G-PQSs (Figure 1), dsRNA viruses showed the most striking difference, as they were most enriched in A-QLS and most depleted in G-PQSs.

The same type of analysis was also performed on individual viral families, to assess potential heterogeneities within Baltimore classes. Indeed, the Polyomaviridae and Poxviridae families, belonging to the first Baltimore class (dsDNA), behaved differently from the other members of the class, having a marked predominance of concordant A-QLSs, with two and three adenine islands within the CDS-containing strand (Supplementary Figures 3, 4). Similarly, the Coronaviridae family was strikingly different from the other members of the fourth Baltimore class (ssRNA+), with the vast majority of A-QLSs present in the opposite strand with respect to CDSs (Supplementary Figures 3, 4).

### Statistical Evidence of the Presence of A Quadruplex-Like Sequences in the Human Viruses’ Genomes

To exclude the possibility that the A-QLS patterns simply correlate with the abundance of A and T nucleotides in viral genomes, we assessed the probability of finding A-QLSs in reshuffled genome sequences with the same length and nucleotide composition of the naturally occurring ones. The evaluation was carried out with two different reshuffling methods, i.e.,(1) at the single nucleotide level, meaning that all bases in the genomes were redistributed individually without constraints and (2) at the tract level, where the number of A tracts of length two, three, and four was maintained identical to the original viral sequences, but the island positions along the genomes were randomly reshuffled. While the first strategy allows evaluating the direct correlation between A-QLS abundance and A/T content, the second method also considers the potential heterogeneous representation of di-, tri-, and tetranucleotide patterns in viral genomes, especially the frequency of A/T homopolymers.

**FIGURE 2:**
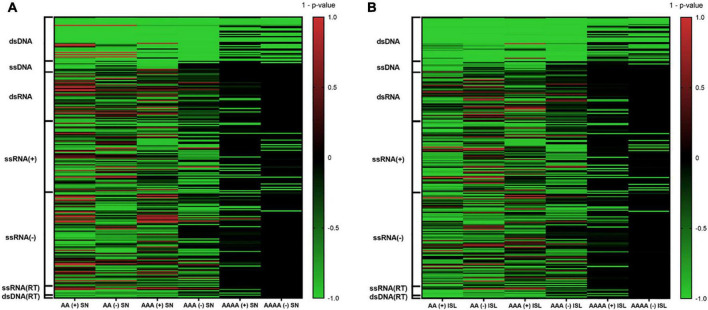
Comparison of A-QLS abundance in real viral genomes vs. simulated genomes reshuffled at **(A)** single nucleotide and **(B)** island levels. Green and red indicate enrichment in real and simulated sequences, respectively.

A total of 10,000 random sequences were generated for each virus and simulation strategy, and all of them were searched for the presence of A-QLSs. Pattern abundance of real viral genomes and simulated sequences was compared and mid *P*-value was calculated as previously reported (Lavezzo et al., 2018). Briefly, a *P*-value close to 0 indicates that the median value of A-QLSs found in real genomes is like that of the simulated ones, whereas *P*-values close to −1 or +1 indicate that the number of A-QLS is significantly enriched in the real or simulated sequences, respectively. The complete results of such comparison are shown as heatmaps in Figure 2, where green and red lines represent enrichment of A-QLSs in the real and reshuffled viral genomes, respectively. Overall, 38% AA, 34.8% AAA, and 20% AAAA QLSs showed a significant difference (*p* < 0.05) between the real and simulated sequences reshuffled at the single nucleotide level. When considering island reshuffling, these values slightly decreased to 36.8, 31.2, and 18%. The complete list of *P*-values is reported in Supplementary Tables 1, 2. Most members of Baltimore classes I, II, and VII (the DNA viruses) showed significantly more A-QLSs in real genomes (green spots in Figure 2) and only some exceptions regarding few members of the dsDNA virus families show an opposite trend (red spots in Figure 2), especially in comparison with simulations reshuffled at the singlenucleotide. Conversely, the RNA classes present more internal variability among their members and between sense and antisense strands.

When considering only the viruses with an A-QLS content significantly different from the reshuffling simulations, we noted that the proportion of those showing a pattern enrichment in real genomes is higher when reshuffling is performed at the island level with respect to singlenucleotide (Supplementary Table 3). This suggests that, during evolution, many human viruses organized their adenines (and thymines or uracils) into homopolymeric tracts more than expected from the raw abundance of these nucleotides, and even more aggregated these islands into clusters.

### Experimental Validation of Selected A Quadruplex-Like Sequence Patterns From Different Viruses

The presence of adjacent As in the same strand, disclosed by bioinformatics analysis, supports the possibility of non-canonical A-, T-, or U-based structure formation. Most of the obtained sequences are characterized by at least four A-islands, each composed by no less than two As: the pattern can be summarized as (A _≥ 2_N_0–12_)_4_, where N indicates any nucleotide in the loop. This nucleotide organization is comparable to that reported for the formation of G4s (Rhodes and Lipps, 2015) and would in principle allow formation of A-tetrads. Noteworthy, the reverse complementary strand of the observed sequences is either T-rich, in DNA viruses, or U-rich in RNA viruses, which could also in principle form tetrads and, possibly, quadruplexes (Gu and Leszczynski, 2001).

**FIGURE 3:**
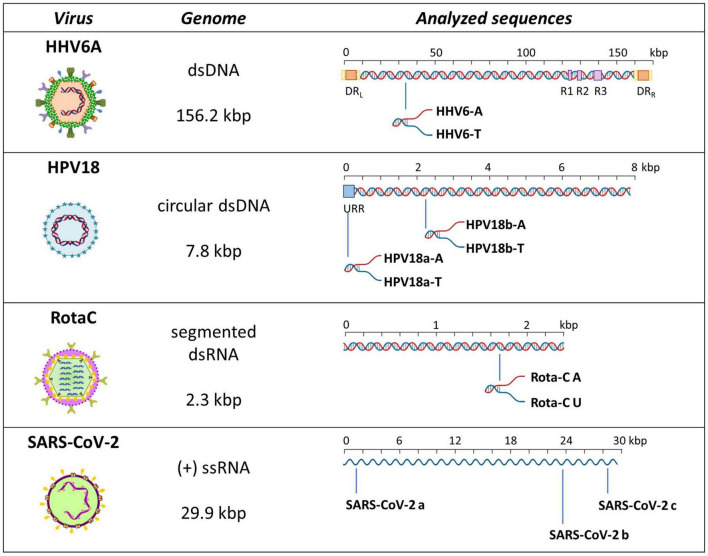
Schematic representation of analyzed sequences. For each sequence, the relative virus, its genome features, and a schematic genome organization are reported. Adapted from Ruggiero and Richter (2018).

To evaluate formation of A-, T-, or U-tetraplexes, we selected representative sequences among those identified by bioinformatics analysis. In particular, we selected putative A-QLSs in the forward and reverse strand of both DNA and RNA viruses, including at least four A-islands and heterogeneous loop length and compositions (Table 1). We selected one sequence from the human herpesvirus 6 type A (HHV6A) and two from the human papillomavirus (HPV), as representative dsDNA viruses, one sequence from Rotavirus C (RotaC) and three from the SARS-CoV-2, as representatives of dsRNA and ssRNA(+) viruses, respectively (Figure 3). For each sequence of HHV6A, HPV, and RotaC, the reverse complementary strand was tested to investigate formation of T and U-based structures. Conservation analysis of selected A-QLSs among different strains of each species was performed as previously reported (Lavezzo et al., 2018), and results are reported in Supplementary Figures 5–8. The analyzed A-QLSs are reported as bars, the height of which represents the scaffold conservation index (SCI), while the x-axis reports the genome location; the local sequence conservation (LSC) is shown as a red line.

**TABLE 1:** List of the selected A-rich sequences and their complementary sequences.

Genome	Name	Sequence (5′-3′)*^a^*
**DNA**	**HHV6A-A**	**AAAAA**CCTG**AAAA**CATCG**AAAAA**CC**AAAAA**
	**HHV6A-T**	**TTTTT**GG**TTTTT**CGATG**TTTT**CAGG**TTTTT**
	**HPV18a-A**	**AAAAAA**GGGAGTAACCG**AAAA**CGGTCGGGACCG**AAAA**CGGTGTATAT**AAAA**
	**HPV18a-T**	**TTTT**ATATACACCG**TTTT**CGGTCCCGACCG**TTTT**CGGTTACTCCC**TTTTTT**
	**HPV18b-A**	**AAAA**TCATTTTT**AAAA**GGAACCCCC**AAAAAAAA**
	**HPV18b-T**	**TTTTTTTT**GGGGGTTCC**TTTT**AAAAATGA**TTTT**
**RNA**	**RotaC-A**	**AAAAAA**UUUAG**AAA**UACU**AAAAU**GUUC**AAAAAA**GCAGC**AAAA**C**AAAA**
	**RotaC-U**	**UUUU**G**UUUU**GCUGC**UUUUUU**GAACA**UUUU**AGUAUUUCUAAA**UUUUUU**
	**SARS-CoV-2a-A**	**AAA**GUUAC**AAAA**GG**AAAA**GCU**AAAAAA**
	**SARS-CoV-2b-A**	**AAA**C**AAA**UUUAC**AAAA**CACCACCAAUU**AAA**
	**SARS-CoV-2c-A**	**AAA**UUGGAUGAC**AAA**GAUCC**AAA**UUUC**AAA**

*^a^Stretches of As, Ts, and Us, able to form tetrads, are reported in bold.*

#### Assessment of Secondary Structures in A-Rich Sequences

**FIGURE 4:**
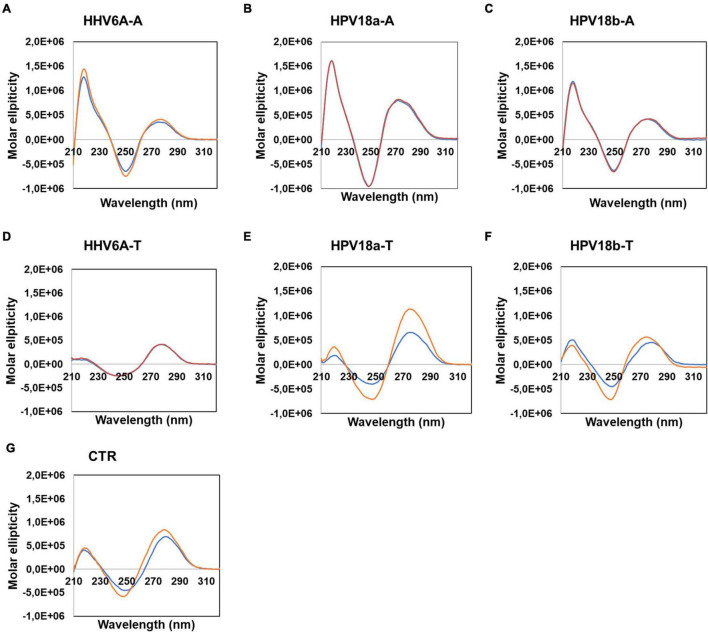
Circular dichroism analysis of HHV6A-A **(A)**, HPV18b-A **(B)**, and HPV-18b-A **(C)**, and respective complementary T-rich sequences **(D–F)**. Samples were prepared in the absence (blue line) or presence (red line) of 100 mM KCl. The CTR sequence **(G)** was used as a control for unstructured DNA. Analysis was performed at 20°C.

To assess non-B secondary conformations of the selected sequences, we initially performed circular dichroism (CD) analysis. CD is a spectrophotometric technique that allows identification of biomolecule secondary structures according to dichroic peaks, which are indicative of the adopted conformation (Kypr et al., 2009). We first performed CD experiments on sequences retrieved from DNA viruses (Figure 4), in particular the human herpesvirus 6 (HHV-6) which is the causative agent of the febrile illness *roseola infantum*, also known as the sixth childhood eruptive disease, which affects almost 100% of the human population (Gilbert-Girard et al., 2017) and the human papilloma virus (HPV) type 18, one of the high-risk HPV subtypes, responsible for more than 70% of cervical cancer worldwide (Doorbar et al., 2015). Samples were prepared in the absence or presence of 100 mM KCl: potassium ions are widely recognized to stabilize G4 structures, by central coordination with the lone pair of electrons from the carbonyl group in position 6 of each G (Spiegel et al., 2019). Analogously, K^+^ has been previously proved to fit in the center of planar A-tetrad using dispersion-corrected density functional theory (DFT)(Van Der Wijst et al., 2010); thus, we employed KCl to observe the possible formation of A-tetrads. A-rich sequences revealed superimposable spectra in both conditions, characterized by two positive peaks at λ ∼ 220 and 280 nm and a negative peak at λ ∼ 250 nm (Figures 4A–C). No changes upon addition of K^+^ were observed. These CD profiles are consistent with poly[d(AT)] DNA, which is characterized by a distinctively narrow minor groove (Alexeev et al., 1987). The complementary T-rich sequences provided slightly different CD spectra (Figures 4D–F), with peaks located at the same wavelength, although with changes in intensities: higher molar ellipticity was observed at λ ∼ 280, with respect to 220 nm. In particular, HHV6A-T showed no difference in the presence/absence of KCl; in contrast, both HPV18-T sequences displayed an increase in molar ellipticity in the presence of KCl, coupled with a mild shift to lower wavelengths. The same behavior was also observed in the CTR sequence (Figure 4G), which is a random DNA control sequence.

**FIGURE 5:**
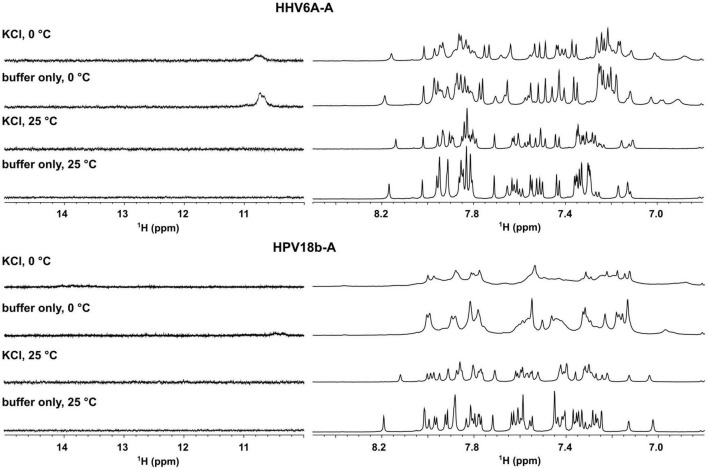
Aromatic (right) and imino (left) region of 1D proton NMR spectra of HHV6A-A, and HPV18b-A oligonucleotides in lithium cacodylate buffer with and without 100 mM KCl. Spectra were recorded at 25 and 0°C.

We next performed nuclear magnetic resonance (NMR) analysis to highlight the presence of any secondary arrangements in selected DNA sequences, namely, HHV6A-A and HPV18b-A. Secondary structures stabilized by Watson-Crick or non-canonical base pairs can be easily recognized, as they give rise to signals in the imino region from δ 9–15 ppm of proton NMR spectra. The test oligonucleotides were prepared in the absence and presence of 100 mM KCl. 1D proton NMR spectra revealed that no signals could be detected in the imino region for the A-rich oligonucleotides before or after addition of KCl. A-rich oligonucleotides are hence in an extended, single-stranded helical form and do not form any type of alternative secondary structure (Figure 5). For HHV6A-A at 25°C, the signals in the aromatic region displayed similar distribution before and after addition of KCl, indicating that no structural rearrangement was induced in the presence of KCl. To detect transient or less stable structures, NMR experiments were repeated at 0°C: a very broad, low-intensity signal appeared at δ 10.8 ppm in the imino region both in the presence and absence of KCl, while distribution of signals in the aromatic region was retained. The similar distribution of signals in the aromatic region observed in both salt and temperature conditions excludes substantial structural changes.

For HPV18b-A, signals in the 1D proton NMR spectrum broadened slightly after salt addition at 25°C. At 0°C, signals in the aromatic region broadened even more, although no new signals could be observed in the imino region.

#### Analysis of Sequence-Induced Polymerase Stop Sites

**FIGURE 6:**
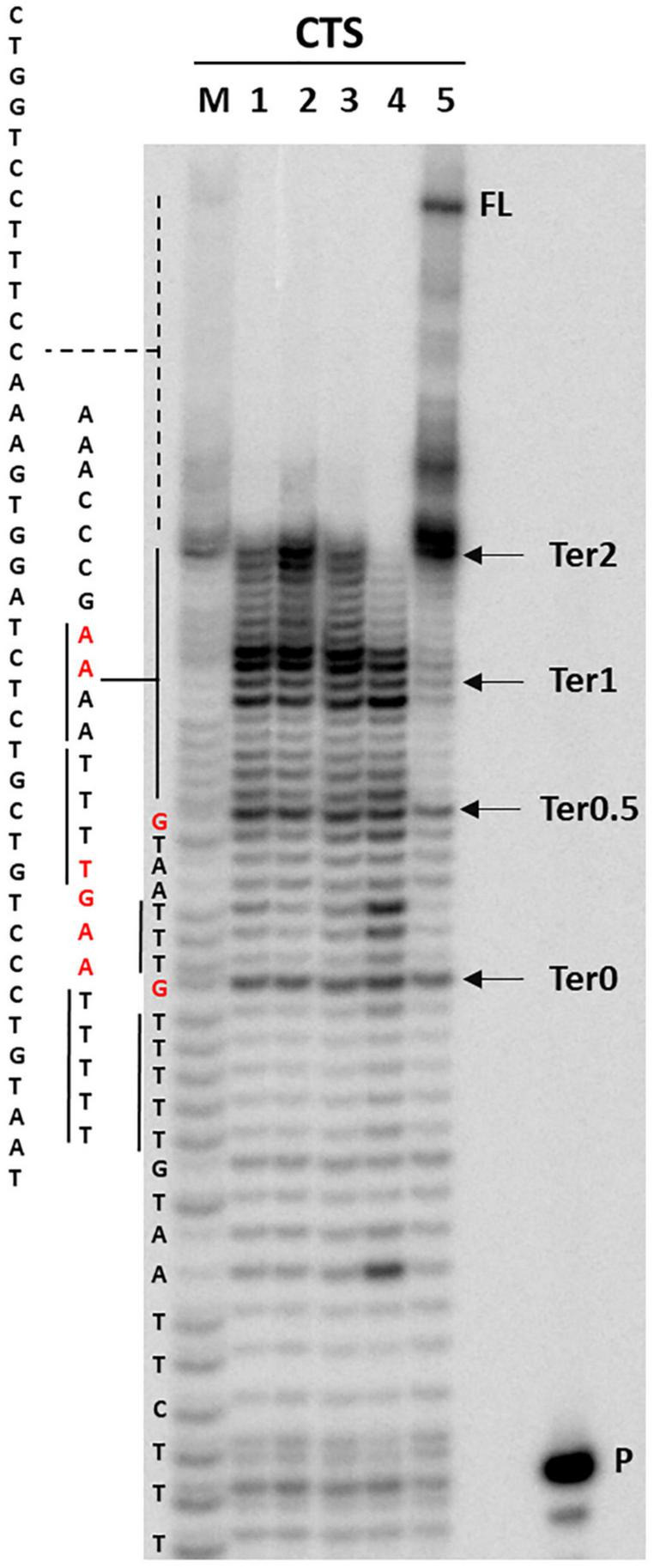
*Taq* polymerase stop assay on HIV-1 CTS sequence. Analysis was performed in the absence (lane 5) or presence of 100 mM different salt types, such a KCl (1), NaCl (2), LiCl (3), and NaF (4). P indicates the unreacted labeled primer; FL indicates the full-length elongation product. Arrows indicate the previously reported termination sites (Lavigne et al., 1997). Bases relative to stop sites are reported in red.

Circular dichroism and nuclear magnetic resonance analysis ruled out the presence of a quadruplex conformation. It has been reported that A tracts may induce DNA to slightly deviate from the standard B-DNA bending (Koo et al., 1986; Koo and Crothers, 1988; De Santis et al., 1990). *Taq* polymerase stop assay has been previously used to prove bending of the HIV-1 CTS sequence, which includes three A-rich sites (Lavigne et al., 1997). We thus performed *Taq* polymerase stop assay on the HIV-1 CTS sequence in different salts to test the best conditions to evaluate oligonucleotide bending, as nucleic acid structures are reported to be highly influenced by the presence of different cations and anions (Meyer and Sühnel, 2003; Bhattacharyya et al., 2016). Based on the reported interaction of A-rich oligonucleotides with different cations and anions (Van Der Wijst et al., 2010), we selected KCl, NaCl, and LiCl salts to test the effect of different monovalent cations, and NaF to test the effect of a different anion. In the absence of additional ions to those required for enzyme activity, full-length elongation product was obtained, with one main stop band at Ter2 and additional milder stops at Ter1, Ter0.5, and Ter0 (Figure 6, lane 5). In saline conditions, no full-length product was detected, and a marked stop band was recovered at Ter1 (lanes 1–4). NaF (lane 4) had the strongest effect on elongation, with additional stops occurring before and after Ter0. Interestingly, the observed stops occurred either within A or T tracts, or within two nucleotides from A or T tracts. The disappearance of the primer band to the same degree in all tested conditions testifies that elongation proceeded similarly in all samples, confirming that the employed saline conditions did not impair polymerase processivity.

We thus next proceeded with other selected representative sequences recovered in our bioinformatics analysis.

**FIGURE 7:**
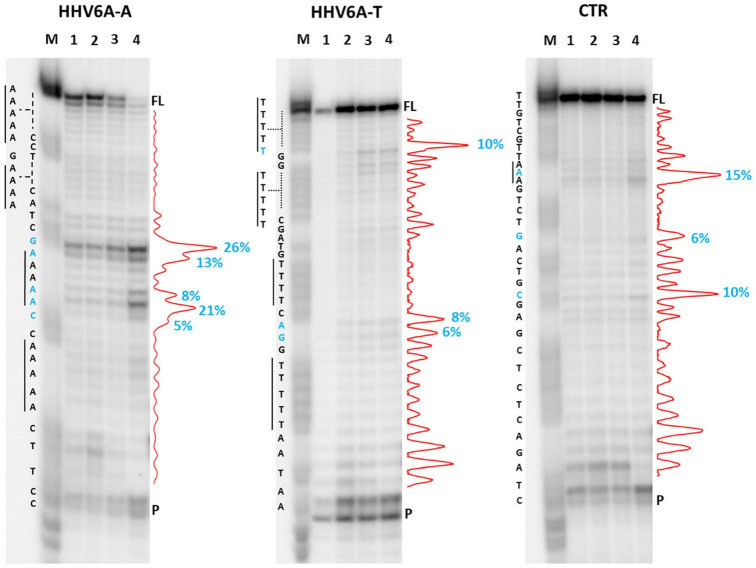
*Taq* polymerase stop assay on HHV6A sequences. Analysis was performed in the presence of 100 mM of different salt types, such as KCl (1), NaCl (2), LiCl (3), and NaF (4). M indicates a sequence marker lane, obtained by the Maxam and Gilbert sequencing protocol; P indicates the unreacted labeled primer; FL indicates the full-length elongation product. Red densitograms show stop bands quantification. Percentages of peaks (≥5%) are reported in blue, along with the corresponding nucleotide. Elongation was performed at 37°C.

We first analyzed a HHV6A A-rich DNA sequence (Table 1), which is included in the coding region of U21 glycoprotein, implicated in immune recognition by cytotoxic T lymphocytes (Figure 3; Hudson et al., 2001). The sequence is characterized by 4 A-tracts composed of 4/5 As separated by short linker sequences (*N* ≤ 5 nt). *Taq* polymerase stop assay was performed on the A-rich sequence HHV6A-A, the reverse T-rich complement sequence HHV6A-T, and a random sequence, unable to fold into predictable non-canonical structures, as control (CTR). For this analysis, a primer annealing sequence and a 5-nucleotide linker corresponding to the naturally occurring sequence were added to the A/T-rich sequences (Supplementary Table 4). In both HHV6A-A and the CTR sequence, NaF induced the most marked stops (94% over the total signal) (Figure 7, lanes 4). Considering single stop sites higher than 5% in intensity over the total stops, in HHV6A-A, all observed stops were either within A tracts or within two nucleotides from A tracts (Figure 7, lanes 1–4, blue bases). In HHV6A-T, stops were mild (up to 33% over the total signal); nonetheless, they again all occurred either within T tracts or within two nucleotides from T tracts. Mild stops were also observed in the CTR sequence (34%): in this case, one stop was within an AAA tract, while the other stops occurred at A/T-unrelated positions.

**FIGURE 8:**
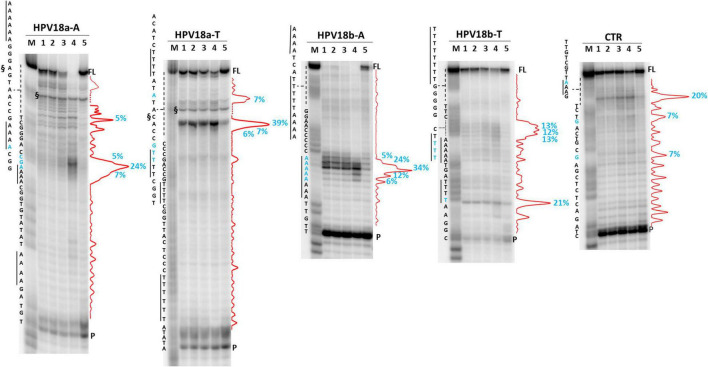
*Taq* polymerase stop assay on HPV-18 sequences. Analysis was performed in the absence (lane 5) or presence of 100 mM different salt types, such as KCl (1), NaCl (2), LiCl (3), and NaF (4). M indicates a sequence marker lane, obtained by the Maxam and Gilbert sequencing protocol; P indicates the unreacted labeled primer; FL indicates the full-length elongation product; § indicates primer dimer band and was excluded from quantitation (dotted line). Red densitograms show stop bands quantification. Percentages of peaks (≥5%) are reported in blue, along with the corresponding nucleotide. Elongation was performed at 37°C.

We next analyzed two HPV A-rich DNA sequences: HPV18a-A is located in HPV upstream regulatory region (URR), which regulates viral-gene transcription and contains the viral origin of replication (Figure 3; McBride and Warburton, 2017); it presents four A-stretches of 4/6 As, separated by long loops (*N* ≥ 10 nt). HPV18b-A is located in the E1 protein coding sequence, the key protein for viral replication initiation (Figure 3; McBride and Warburton, 2017); it contains four AAAAs runs, separated by loops of 8, 9, and 0 nucleotides. We performed analysis on A-rich HPV18a-A and HPV18b-A sequences, their T-rich reverse complement, HPV18a-T and HPV18b-T, and the control CTR (Figure 8). In the A-rich sequences, we observed enhanced stop sites in NaF (98% in HPV18a-A and 91% in HPV18b-A over the total signal) (lanes 4), while less intense sites were also present in the other salt conditions; in T-rich sequences, all stop sites were similar among the different salts (75% in HPV18a-T and 57% in HPV18b-T over the total signal), but one site in HPV18b-T corresponded to a stretch of A and was enhanced in NaF (lanes 4). In HPV18a-A and HPV18a-T 4 over 4 and 3 over 4 recovered stop sites, respectively, corresponded to A/T tracts or were within two bases from A/T tracts. In HPV18b-A and HPV18b-T, all retrieved sites were within A/T tracts or within two bases from A/T tracts.

**FIGURE 9:**
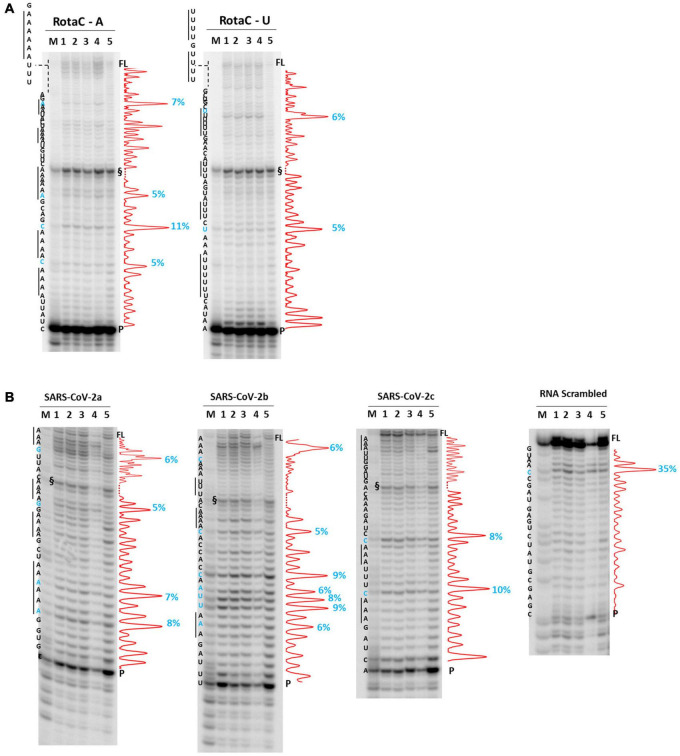
*RT* stop assay on RotaC **(A)** and SARS-CoV-2 **(B)** sequences. Analysis was performed in the absence (lane 5) or presence of 100 mM different salt types, such as KCl (1), NaCl (2), LiCl (3), and NaF (4). M indicates a sequence marker lane, obtained by the Maxam and Gilbert sequencing protocol; P indicates the unreacted labeled primer; FL indicates the full-length elongation product; § indicates primer dimer band and was excluded from quantitation (dotted line). Red densitograms show stop bands quantification. Percentages of peaks (≥5%) are reported in blue, along with the corresponding nucleotide. Elongation was performed at 44°C.

We next moved to RNA viruses and selected sequences derived from RotaC and SARS-CoV-2 viruses. Rotaviruses belong to one of the two families of viruses that are part of the dsRNA viruses, which showed the highest content of A-QLSs, incidentally clustered in the concordant strand (Figure 1A). Rotaviruses are responsible for severe, dehydrating infantile gastroenteritis that are life-threatening for children under age five living in low-income countries (Crawford et al., 2017). We selected a sequence (RotaC-A), which presents six A-tracts, ranging from 3 to 6 As, separated by short loops (*N* ≤ 5 nt), and located in the coding region of the viral protein 4 (VP4), an outer capsid protein involved in the regulation of viral entry, hence responsible for rotavirus virulence (Figure 4; Nejmeddine et al., 2000). We performed the analysis on RotaC-A and its complementary U-rich strand RotaC-U (Figure 9A). From SARS-CoV-2, the virus responsible for the latest pandemic and one of the most challenging crisis for humanity in the recent years, which led to over six million deaths from December 2019 to March 2022 (WHO, 2022), we selected three sequences, SARS-CoV-2a, SARS-CoV-2b, and SARS-CoV-2c (Table 1 and Figure 3). Sequence SARS-CoV-2a is located in the orf1ab (SARS-CoV-2a), which encodes for several non-structural proteins, including the RNA-dependent RNA polymerase. This sequence is formed by four A-tracts, including 3, 4, and 6 As, separated by short loops. Sequence SARS-CoV-2b was retrieved from the coding sequence of gene S, coding for the spike surface glycoprotein, which mediates virus attachment to the human angiotensin-converting enzyme (ACE) 2 receptor, while sequence SARS-CoV-2c derives from the N gene, encoding for the nucleocapsid protein, which tightly binds and protects viral RNA (Kumar et al., 2020). Sequences SARS-CoV-2b and SARS-CoV-2c include A-tracts formed by three or four As, separated by short and long loops. In the case of SARS-CoV-2, we tested only the A-rich sequences (Figure 9B). The RNA sequences were processed by stop assay employing the HIV-1 reverse transcriptase (RT) and a scrambled RNA was used as control. The RNA template was obtained through *in vitro* transcription, starting from a complementary synthetic DNA template (Supplementary Table 4), which included the T7 promoter site. In general, the elongation reaction with RT was less efficient than with *Taq* polymerase, and less full-length products were observed (Figure 9).

Differently from the DNA templates, we did not observe enhanced stop sites in the presence of NaF salt. In RotaC, the intensity of the stop sites was similar between the A-rich (68% over the total signal) and U-rich (74% over the total signal) sequence. Like the DNA templates, sites were within A/U tracts or within two bases from A/U tracts (4 over 4 total sites in RotaC-A and 2 over 2 total sites in RotaC-U). Similarly, in the A-rich SARS-CoV-2 sequences (72, 83, and 68% over the total signal in SARS-a, SARS-b, and SARS-c, respectively), most stop sites had the same location with respect to A/U tracts (4 over 4 total sites in SARS-CoV-2a, 5 over 7 total sites in SARS-CoV-2b, 2 over 2 total sites in SARS-CoV-2c). In contrast, in the CTR sequence, the most significant recovered stop site was at a different location.

A summary of the identified stop sites within viral templates is presented in Table 2.

**TABLE 2:** Summary of identified stop sites within viral templates.

Genome	Name	Sequence (5′-3′)*^a^*
**DNA**	**HHV6A-A**	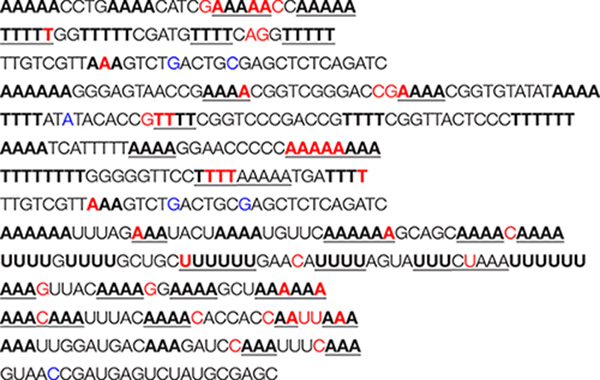
	**HHV6A-T**	
	**CTR**	
	**HPV18a-A**	
	**HPV18a-T**	
	**HPV18b-A**	
	**HPV18b-T**	
	**CTR**	
**RNA**	**RotaC-A**	
	**RotaC-U**	
	**SARS-CoV-2a-A**	
	**SARS-CoV-2b-A**	
	**SARS-CoV-2c-A**	
	**CTR**	

*^a^In bold: A, T, U tracts. In red: bases in Taq pol stop assays in Figures 7–9 that are within or within two bases from A, T, U tracts. In blue: bases in Taq pol stop assays in Figures 7–9 that are outside two bases from A, T, U tracts. Underlined bases are A, T, U tracts involved in stops (i.e., stops within the tract or within two bases from the tract).*

## Discussion

We have shown here that virus genomes are enriched in A/T/U-tracts that in principle could fold into a quadruplex according to quadruplex folding rules. Our investigation showed that quadruplexes do not form, but A/T/U tracts pause polymerase progression.

A-tracts have been reported in the genomes of both prokaryotic and eukaryotic organisms, including humans (Koo et al., 1986; Koo and Crothers, 1988; Behe, 1995; Dechering et al., 1998; Tolstorukov et al., 2005; Fire et al., 2006; Haran and Mohanty, 2009; Marin-Gonzalez et al., 2020). A-tracts within DNA double helix show a peculiar conformation with a characteristic narrow minor groove (Nelson et al., 1987; Sherer et al., 1999). When two or more A-tracts are present, they induce a significant global curvature of the DNA helical axis (Koo et al., 1986; Haran and Mohanty, 2009). Both A-tract curvature and their molecular conformation are suggested to have biological relevance (Hagerman, 1990; Haran and Mohanty, 2009; Segal and Widom, 2009; Rohs et al., 2010): in eukaryotic genomes, A-tract distribution is essential in nucleosome organization and transcription regulation (Kaplan et al., 2009; Rohs et al., 2009; Raveh-Sadka et al., 2012) and A-tracts are implicated in recombination (Hagerman, 1990; Haran and Mohanty, 2009), replication (Haran and Mohanty, 2009), and immune response to viruses (Ablasser et al., 2009; Chiu et al., 2009). A-tracts are reported to stabilize DNA tertiary structures, such as loops and supercoils (Johnson et al., 2013), and can be recognized by proteins to achieve binding specificity (Rohs et al., 2010). In microorganisms, A-tracts are involved in promoting DNA looping in *Klebsiella pneumoniae*, in favoring RNA polymerase interaction with the UP promoter in bacteria, and in terminating DNA synthesis in HIV-1 replication, by promoting reverse transcriptase (RT) displacement (Haran and Mohanty, 2009).

Here, we reported for the first time that A-tracts are widespread in virus genomes as well. This evidence suggests that viruses may exploit similar A-tractmediated mechanisms as those reported for the eukaryotic cell.

We have shown that the presence of A-tracts is related to polymerase pausing, which occurred within the A-tract or at the most at two bases of distance from it, indicating the specificity of this effect (Table 2). A-tracts have been reported to be sensitive to salts, in particular to Na^+^ ions (De Santis et al., 1990). In our case, all salts increased overall polymerase pausing. At the DNA level, NaF but not NaCl further increased polymerase pausing: we ascribed this behavior to a toxic effect on the enzyme by the F-ion, which makes the enzyme less efficient and thus more prone to stop at critical sites. At the RNA level, this effect was not observed likely because of the different impact of NaF on the RT enzyme.

Also, T and U tracts triggered polymerase pausing: we ascribed this effect to a structural effect of the T/U-rich template strand or else to the A-induced bending of the newly synthesized strand that could impact on enzyme activity. Generally, however, A-tracts on the template strand show the highest impact on polymerase activity.

In single-stranded nucleic acids (both in the virus DNA/RNA genome and in mRNA), “A” bases tend to stack on each other in A-tracts (Bloomfield et al., 2000; McIntosh et al., 2014; Ou et al., 2020): these structures in eukaryotes have been reported mainly but not only at the 3′-end of mRNA and shown to be implicated in the regulation of protein expression (Kojima et al., 2012) and to further serve for protein recognition (Theobald et al., 2003). Also in virus HIV-1, A-tracts were reported to function as recognition signals for interacting agents, such as proteins or enzymes (Lavigne et al., 1997). In fact, A-proteins interaction has been found to be an ancient mechanism, refined throughout evolution (Narunsky et al., 2020).

Interestingly, most of the identified A-tracts were in the coding strand (i.e., the strand that corresponds to mRNA both at the DNA and RNA level), suggesting that they could be relevant during genome replication of RNA viruses and translation of DNA and RNA viruses. Viruses generally produce polyproteins from polycistronic mRNA and exploit mechanisms such as reinitiation of translation, leaky scanning, suppression of termination, and ribosomal frameshifting (Jaafar and Kieft, 2019) that benefit from polymerase pausing. Thus, A-tracts, by slowing down polymerase progression, could aid in the translation processing of viral mRNAs.

In addition, the bias between coding and non-coding strand was particularly evident in RNA viruses compared with large genome DNA viruses. RNA viruses generally perform their complete life cycle in the cytoplasmatic compartment: a potential explanation is that, as they do not have access to the nuclear proteome and to the many regulators of transcription normally available to DNA viruses, they had to maximally develop and exploit alternative mechanisms based on nucleic acid epigenetic signals.

Statistical analysis of A-tracts distribution in virus genomes support a specific biological role for A-tracts in viruses. Conservation analysis performed on selected genomes revealed that, especially in HPV and RotaC viruses, which belong to Baltimore classes I and VII, respectively, A-tracts were highly conserved (above 90%). Given that viruses, especially RNA viruses, are characterized by remarkable mutation rates, these data *per se* support the relevance of A-tracts in the virus biology. Interestingly, HPV and RotaC viruses were the least enriched in putative G4-forming sequences (Lavezzo et al., 2018): it is therefore tempting to speculate that A-tracts complement or fill in a biological role that in other viruses is provided by G4s.

Despite the great advances achieved in the antiviral field in the past years, most of the available therapies do not provide virus eradication or sustained virological response; therefore, the identification of innovative antiviral targets is urged. A-tracts and their possible interaction with proteins may reveal new mechanisms in virus biology and possibly new antiviral targets.

## Materials and Methods

### Putative Quadruplex Forming Sequence Detection and Evaluation of Conservation

All the analyzed genomes of viral species were retrieved from http://viralzone.expasy.org/all_by_species/678.html (accessed on April, 2016) and downloaded from GenBank, according to Lavezzo et al. (2018). For each species, a reference genome was chosen from all available genomes, and the corresponding GFF containing genome annotation was downloaded. Multiple alignments were built for every species with unsearch8 (Edgar, 2010), using an identity threshold (60%) to account for viral variability. These multiple alignments were employed to search A-QLS using QPARSE (Berselli et al., 2020). This tool allowed reporting a A-QLS when at least four consecutive adenine islands (A-islands) were detected. The maximum loop distance (-L parameter) searched between two consecutive A-islands was 7 for A-islands with two As and 12 for A-island with three or four As. The length of the islands is determined by the -m option. Three combinations of parameters were applied: *m* = 2 and *L* = 7; *m* = 3 and *L* = 12; *m* = 4 and *L* = 12. A-QLSs were also searched in the negative strands by looking for Ts, instead of As. Concordant, discordant, and non-coding A-QLSs were determined according to the strand of CDSs as present in the GFF files.

The conservation of each A-QLS in the multiple aligned genomes of the viruses was revealed by looking at the conservation of the amount of As and connecting loops between two A-islands. Then, different indexes were calculated to measure the nucleotide sequence conservation of viral genomes and the A-QLSs:

1. *A4_scaffold_conservation_index* (A4_SCI): this index calculates the percentage of independent genomes that maintain a corresponding A-island found in every A-QLS detected.


$$A\,4_{S\,C\,I}=\frac{N_{A_{i\,s\,l\,a\,n\,d\,s}}}{N_{t\,o\,t}}*100$$

2. *Loop_conservation*: this index is the difference between A4_SCI and the local conservation of the viral sequence that cover the A-QLS (LSC_*A4*_):


$$L\,o\,o\,p_{c\,o\,n\,s\,e\,r\,v\,a\,t\,i\,o\,n}=A\,4_{S\,C\,I}-L\,S\,C_{A\,4}$$

LSC_*A4*_ is calculated as the average of LSC windows overlapping the A-QLS. The measure of LSC is calculated by a sliding window of fixed length (length 20, shift 10), which averages the conservation values of each position. All positions are extracted from the multiple sequence alignments with Jalview (Waterhouse et al., 2009). These are formally defined as:


$$L\,S\,C=\frac{\sum_{i=1}^{20}c_{m\,a\,x\,i}}{20}$$

$$L\,S\,C_{A\,4}=\frac{L\,S\,C_{1}+\ldots +L\,S\,C_{n}}{n}$$

where*c*_*maxi*_ is the maximum conservation at position i of the multiple aligned sequences and *n* is the number of windows overlapping the A-QLS.

The results of these analyses are presented individually for four viral species: HHV6A, HPV18, RotaC, and SARS-CoV-2, as shown earlier. All charts were generated with Plotly (https://plot.ly), exploiting Pandas (McKinney, 2010) and Numpy Python (Walt et al., 2011) libraries. Multiple alignments are visualized with MSAViewer (Yachdav et al., 2016) and genomic features with JBrowse 1.15.0 (Skinner et al., 2009). Unless otherwise stated, analyses were conducted with in-house developed Python and Perl scripts.

### Evaluation of A Quadruplex-Like Sequences Conservation in Real vs. Randomized Viral Sequences

The significance of A-QLSs abundance in viruses was assessed by simulating viral genomes. These simulations were generated by reshuffling the same number of nucleotides present in the real data, but with different criteria. For each virus, we generated a string of length *n* made of only “Z” characters, with *n* corresponding to the length of the virus reference genome; then, randomly, an amount of Zs equal to the number of A-, AA-, AAA-, AAAA-, T-, TT-, TTT-, TTTT-islands found in the reference sequence was removed and replaced with A or T islands, respectively. Accordingly, 10,000 independent sequences were generated for four different simulated datasets, one for each island length.

### Statistical Methods

The simulated sequences were examined for the presence of A-QLS as described above. The 10,000 counts obtained for each simulation formed the empirical distribution for A-QLS prevalence under the hypothesis of random assembling of the genome in the different reshuffling models. The mid*P*-value was calculated using an *ex novo* function:


$$mid-Pvalue=P\left(T>T\right)+\frac{1}{2}P\left(T=T\right)$$

where T* is the total number of islands found in the reference genome and T is a vector containing separately all the islands found for each independent sequence.

### Experimental Validation of Selected A-Putative Quadruplex Forming Sequences

All oligonucleotides used in this study were purchased from Sigma Aldrich (Supplementary Table 4). Oligos were previously resuspended in TE (Tris 1 M, pH 8, EDTA 0.5 M, pH 8) to a final concentration of 1 mM and were properly diluted to perform the experiment.

### Circular Dichroism

Oligonucleotides were diluted to a final concentration of 3 μM in lithium cacodylate buffer (10 mM, pH 7.4) in the presence or absence of salts (100 mM), as indicated. After annealing (5 min at 95°C), samples were gradually cooled to room temperature to let them acquire their natural conformation and, where specified, compounds were added at a final concentration of 12 μM. CD spectra were measured after 24 h using Chirascan-Plus (Applied Photophysics, Leatherhead, United Kingdom) equipped with a Peltier temperature controller. Samples were tested in a quartz cell of 5 mm optical path length and over a wavelength range of 210–320 nm. All spectra were baseline-corrected for signal contribution due to the buffer and the observed ellipticities were converted to mean residue ellipticity (θ) = deg × cm^2^ × dmol^–1^ (mol ellip).

### Nuclear Magnetic Resonance

Oligonucleotides for NMR measurements were synthesized on a K&A Laborgeraete GbR DNA/RNA Synthesizer H-8 using standard phosphoramidite chemistry in DMT-on mode. Oligonucleotides were cleaved from support and deprotected with 1:1 mixture of methylamine and ammonium hydroxide. Glen-Pak cartridges in reverse-phase mode were used to remove non-full-length abortive sequences. Oligonucleotides were further desalted with FPLC, dried on lyophilizer, and dissolved in water. Samples were prepared at 0.1 mM oligonucleotide concentration in 10 mM lithium cacodylate buffer, pH 7.4, with or without addition of 100 mM KCl, annealed for 5 min at 95°C, and slowly cooled to room temperature overnight.

Nuclear magnetic resonance experiments were performed at 25 and 0°C on an Agilent NMR Systems 600 MHz spectrometer with 5 mm 1H-optimized triple-resonance cryo probe. Spectra were processed and visualized with TopSpin 4.08 (Lee et al., 2015).

### Taq Polymerase Stop Assay

The *Taq* primer (Supplementary Table 4) was 5′-end labeled with [γ-^32^P]-ATP using T4 polynucleotide kinase at 37°C for 30 min and then purified with G-25 column (MicroSpin G-25 columns, GE Healthcare). Once labeled, the primer (10 nM) was annealed to the appropriate template (36 nM) in lithium cacodylate buffer (10 mM, pH 7.4) at 95°C for 5 min and different salts (e.g., KCl, LiCl, NaCl, NaF, CaCl_2_, and MgCl_2_) were added at a final concentration of 100 mM, as indicated.

Elongation was subsequently carried out at 37°C for 30 min using Ampli*Taq* Gold DNA polymerase (2U/reaction, Applied Biosystem, Waltham, United States) and all the reactions were stopped by EtOH precipitation. The extension products were separated on a 16% denaturing gel and visualized by phosphorimaging (Typhoon FLA 9000, GE Healthcare, Chicago, United States). Markers were prepared according to the Maxam and Gilbert sequencing protocol (Maxam and Gilbert, 1980) using the labeled elongation product as a template. Briefly, elongation products were treated for 5 min with formic acid and reactions were blocked through EtOH precipitation. Samples were then cleaved with piperidine 1 M for 30 min at 90°C and concentrated in SpeedVac, before loading onto the gel.

### Reverse Transcriptase Stop Assay

The 5′-end labeled RT primer (Supplementary Table 4) (20 μM) was annealed to the template (2.5 μM) in lithium cacodylate buffer (10 mM, pH 7.4) at 95°C for 5 min. The primer extension reaction was performed using recombinant HIV-1 Reverse Transcriptase (1 U/reaction, Calbiochem, San Diego, United States) at 44°C for 1 h. Reaction products were treated with NaOH (2 N) at 95°C for 3 min for residual RNA hydrolysis and pH was neutralized with HCl (2 N). The processing of samples and the visualization of the gel were carried out as described in *Taq* polymerase stop assay.

### *In-vitro* Transcription

*In vitro* transcription was performed using TranscriptAid T7 High Yield Transcription kit (Thermo Scientific, Waltham, United States). The RNA templates (2 μg, Supplementary Table 4) were incubated for 6 h at 37°C with enzyme mix, including T7 RNA polymerase and RNase inhibitors (Thermo Scientific RiboLock RNase). After RNase-free DNase treatment, the obtained RNA was purified by phenol-chloroform extraction, precipitated with EtOH, resuspended in DEPC-H_2_O, and stored at −80°C.

## Data Availability Statement

The datasets presented in this study can be found in online repositories. The names of the repository/repositories and accession number(s) can be found below: Zenodo, https://doi.org/10.5281/zenodo.6442530.

## Author Contributions

SR and ST conceived the work. ER, EL, IZ, MG, and MM performed the experiments. ER, EL, and SR wrote the manuscript. JP, ST, and SR revised the manuscript. All authors have given approval to the final version of the manuscript.

## Conflict of Interest

The authors declare that the research was conducted in the absence of any commercial or financial relationships that could be construed as a potential conflict of interest.

## Publisher’s Note

All claims expressed in this article are solely those of the authors and do not necessarily represent those of their affiliated organizations, or those of the publisher, the editors and the reviewers. Any product that may be evaluated in this article, or claim that may be made by its manufacturer, is not guaranteed or endorsed by the publisher.
