# Supplementary material for: Human Virus Genomes Are Enriched in Conserved Adenine/Thymine/Uracil Multiple Tracts That Pause Polymerase Progression
Source: Front Microbiol. 2022 Jun 1;13:915069. doi: 10.3389/fmicb.2022.915069 (PMC9198555; doi:10.3389/fmicb.2022.915069)
Supplement: Supplementary file 1 [file Table_1.DOCX]

Supplementary Material

Attached Excel files include the MID_P_VALUE of comparison between real and simulated viral genomes (Table S1 single nucleotide reshuffling, Table S2 island reshuffling)

**
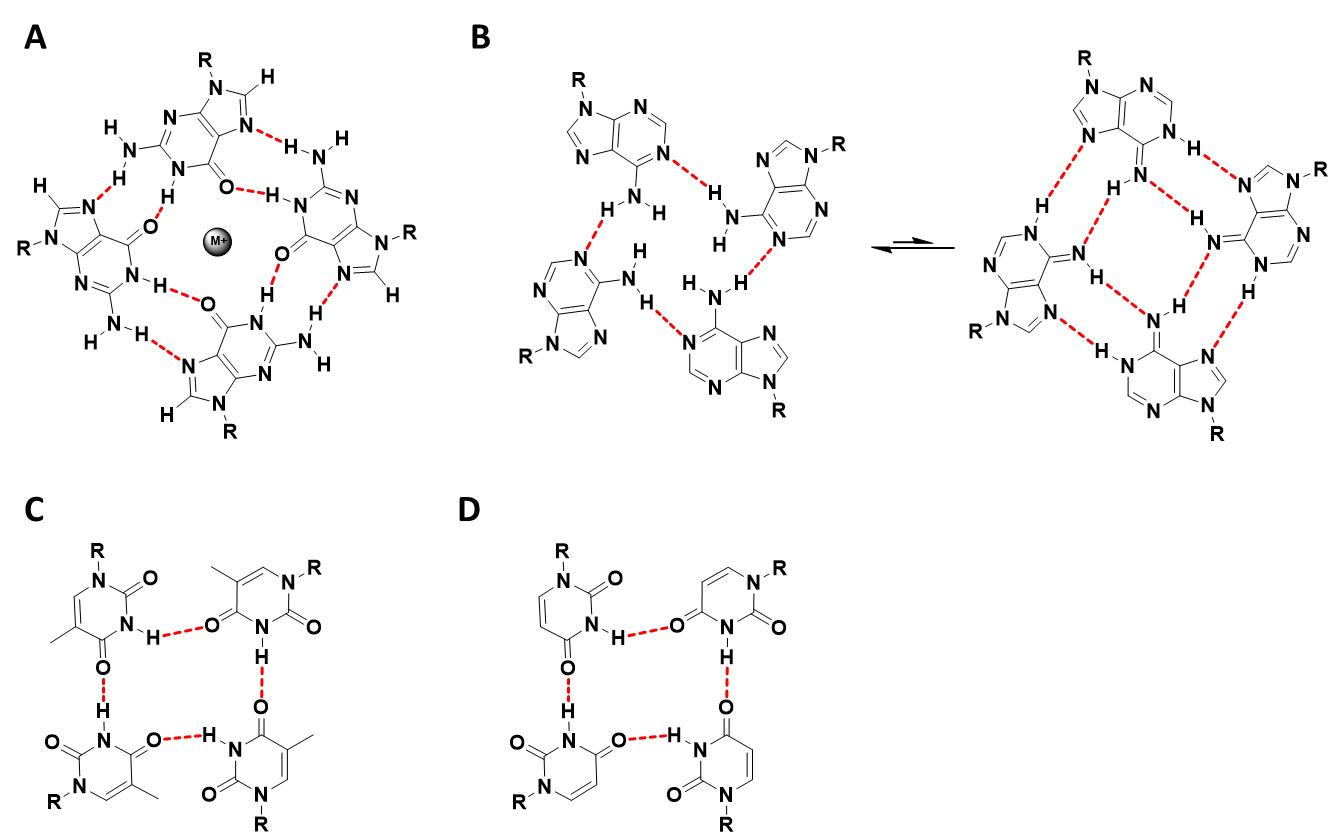
**

**Figure S1. Nucleobases tetrad representative chemical structures.** Purine-based tetrads: (A) Chemical structure of a G-tetrad formed by four Gs coordinated by a monovalent cation (M+); (B) Chemical structure of the two tautomeric forms of A-quartets composed of four As linked together through four or eight H-bonds. Pyrimidine-based tetrads: (C) thymine, (D) uracil.

**
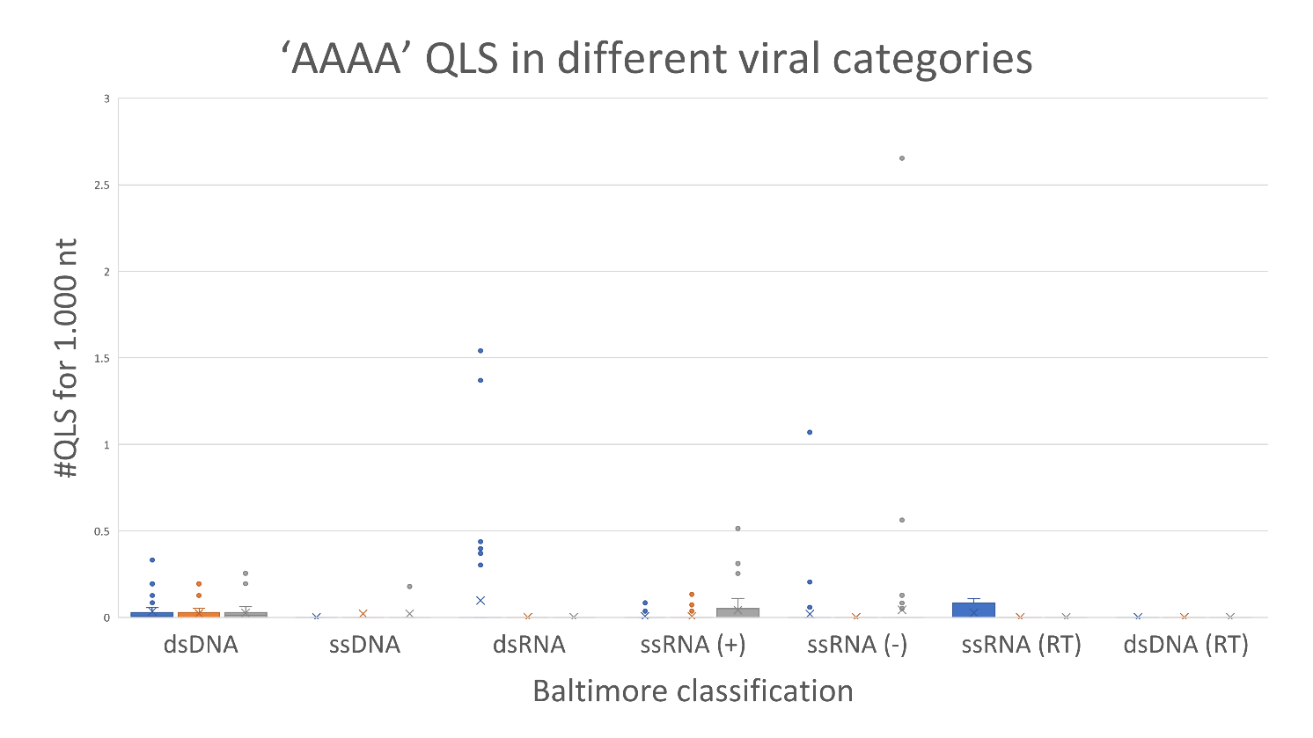

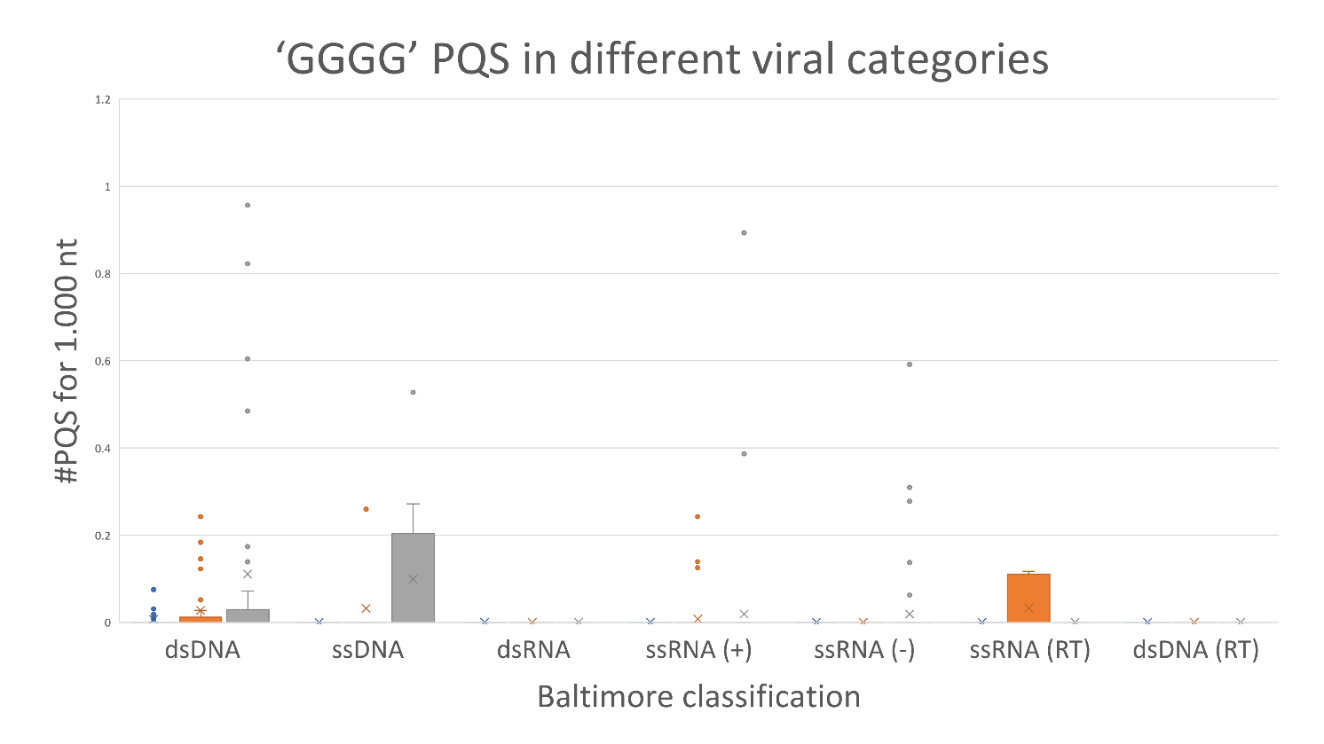
**

**Figure S2.** Normalized abundance of A-QLSs (A) and G-PQSs (B) in the genome of all human viruses, grouped by Baltimore class. Each panel refers to the specified type of island (AAAA for A-QLSs and GGGG for G-PQSs); boxplots are delimited by the first and third quartile and the straight line drawn inside is the median value of the QLS distribution. Whiskers delimit all the points that fall above/below the third/first quartile plus/minus 1.5 times the interquartile range (IQR). Blue, orange, and gray box plots indicate the strand where the predicted patterns are found with respect to coding sequences and refer to concordant (same strand of a CDS), discordant (opposite strand of a CDS), and non-coding (no CDS overlaps that pattern), respectively.


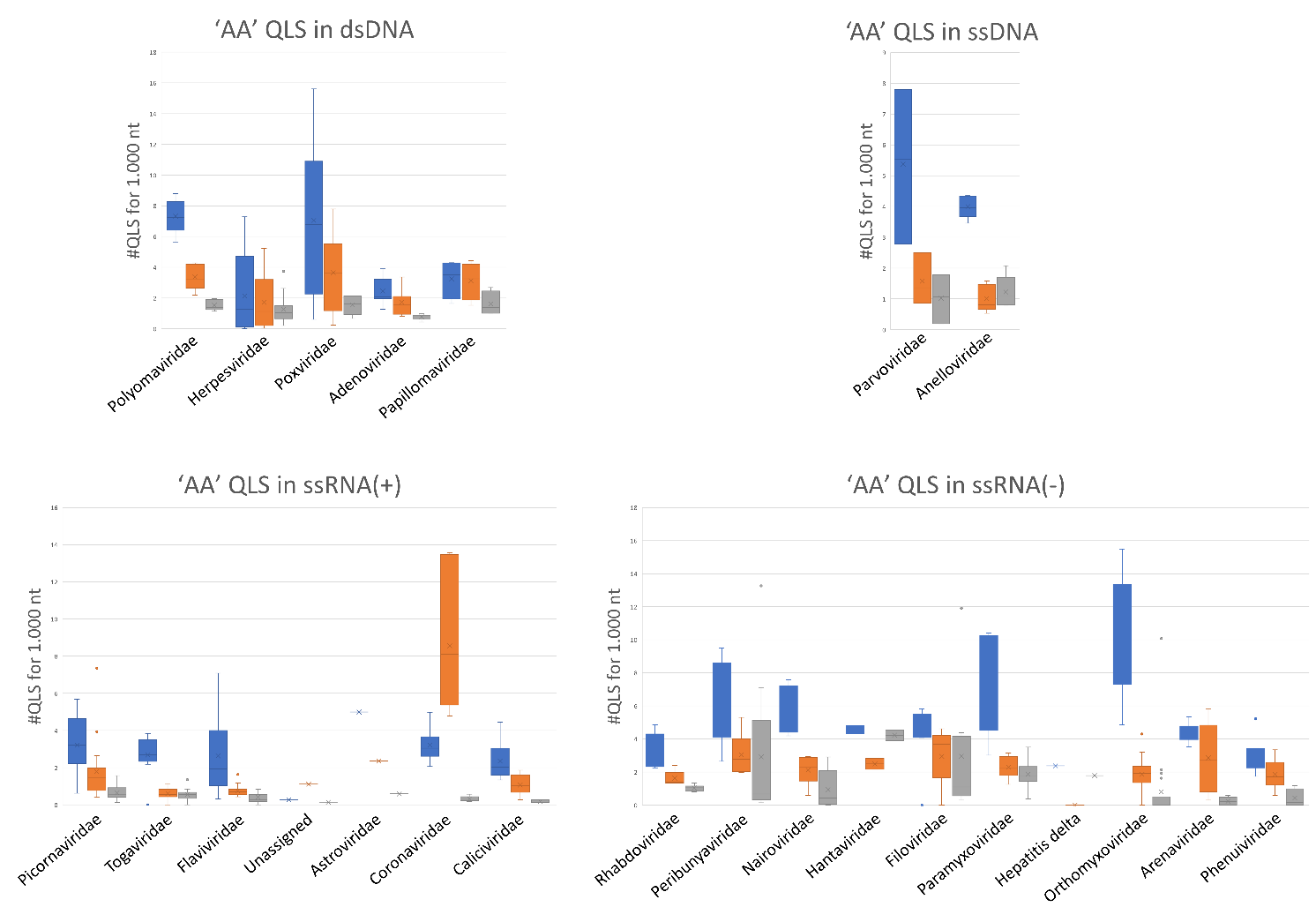


**Figure S3.** Normalized abundance of A-QLSs with islands of length two in the genome of human viruses belonging to the first, second, fourth, and fifth Baltimore classes and grouped by family. Boxplots are delimited by the first and third quartile and the straight line drawn inside is the median value of the QLS distribution. Whiskers delimit all the points that fall above/below the third/first quartile plus/minus 1.5 times the interquartile range (IQR). Blue, orange, and gray box plots indicate the strand where the predicted patterns are found with respect to coding sequences and refer to concordant (same strand of a CDS), discordant (opposite strand of a CDS), and non-coding (no CDS overlaps that pattern), respectively.

**
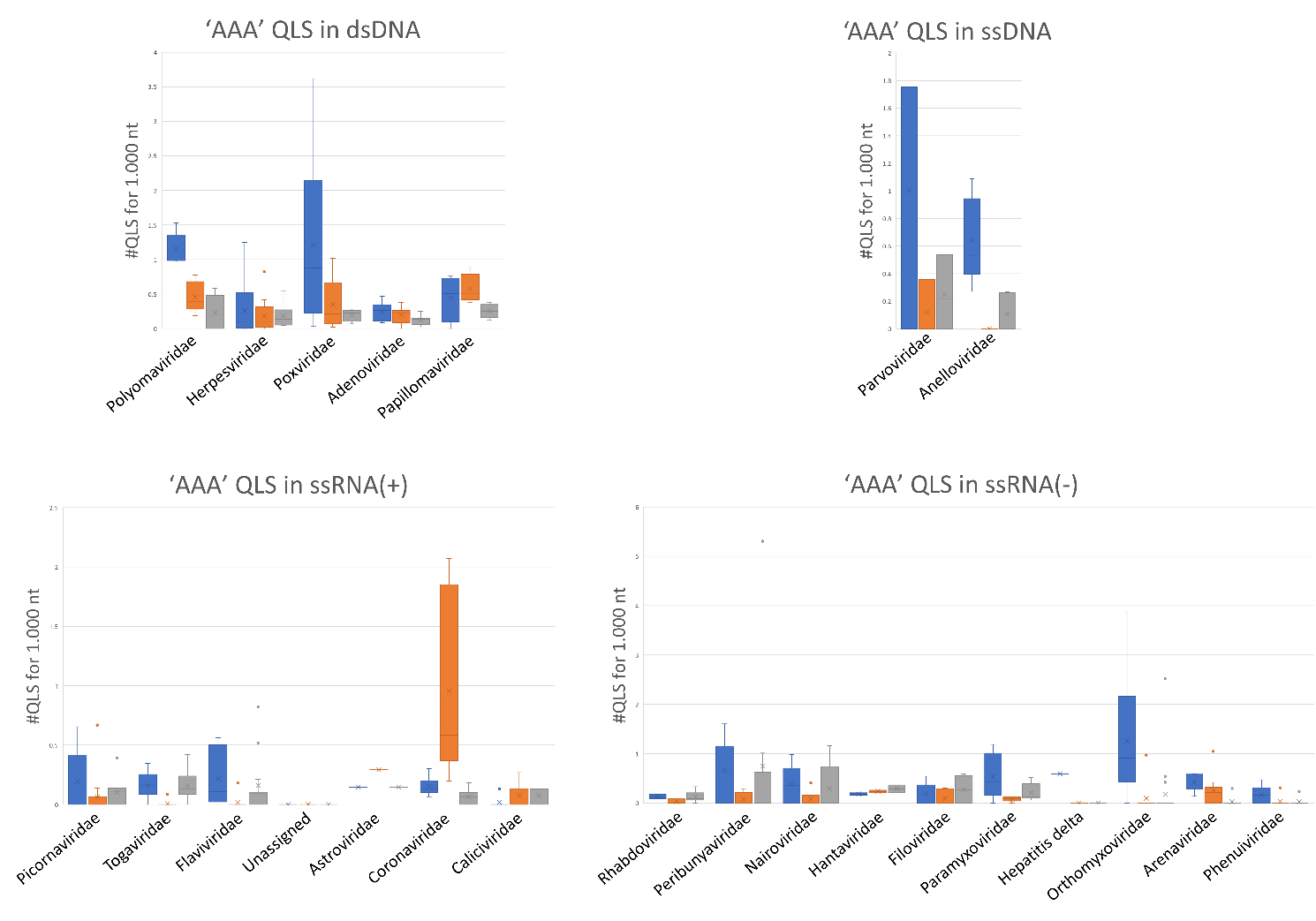
**

**Figure S4.** Normalized abundance of A-QLSs with islands of length three in the genome of human viruses belonging to the first, second, fourth, and fifth Baltimore classes and grouped by family. Boxplots are delimited by the first and third quartile and the straight line drawn inside is the median value of the QLS distribution. Whiskers delimit all the points that fall above/below the third/first quartile plus/minus 1.5 times the interquartile range (IQR). Blue, orange, and gray box plots indicate the strand where the predicted patterns are found with respect to coding sequences and refer to concordant (same strand of a CDS), discordant (opposite strand of a CDS), and non-coding (no CDS overlaps that pattern), respectively.

**Conservation analysis**

**
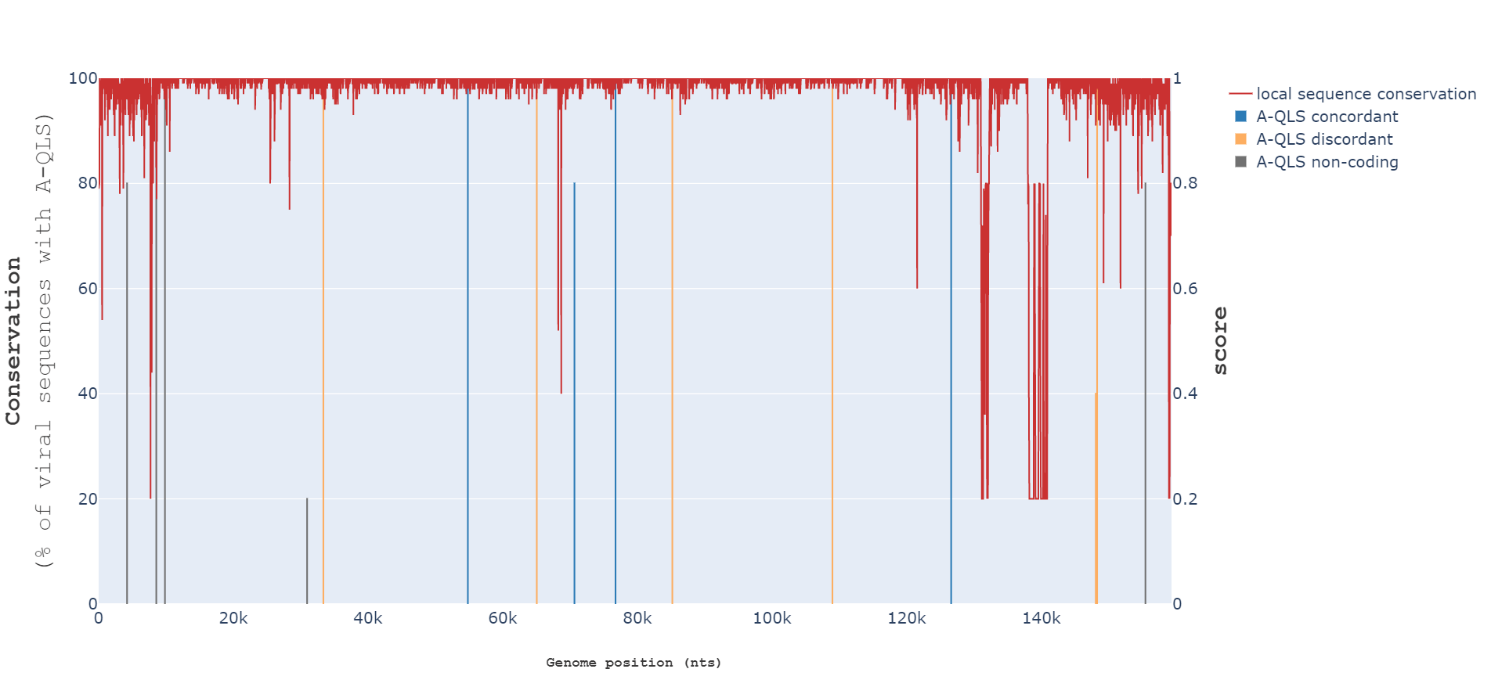
Fig S5. Conservation of A-QLSs in the HHV-6A genome.** Concordant, discordant, and non-coding A-QLSs are indicated as blue, orange, and grey vertical bars, respectively. Local sequence conservation (LSC) of viral genomes is shown as a red broken line. The x-axis indicates genome position, the y-axis the conservation %.


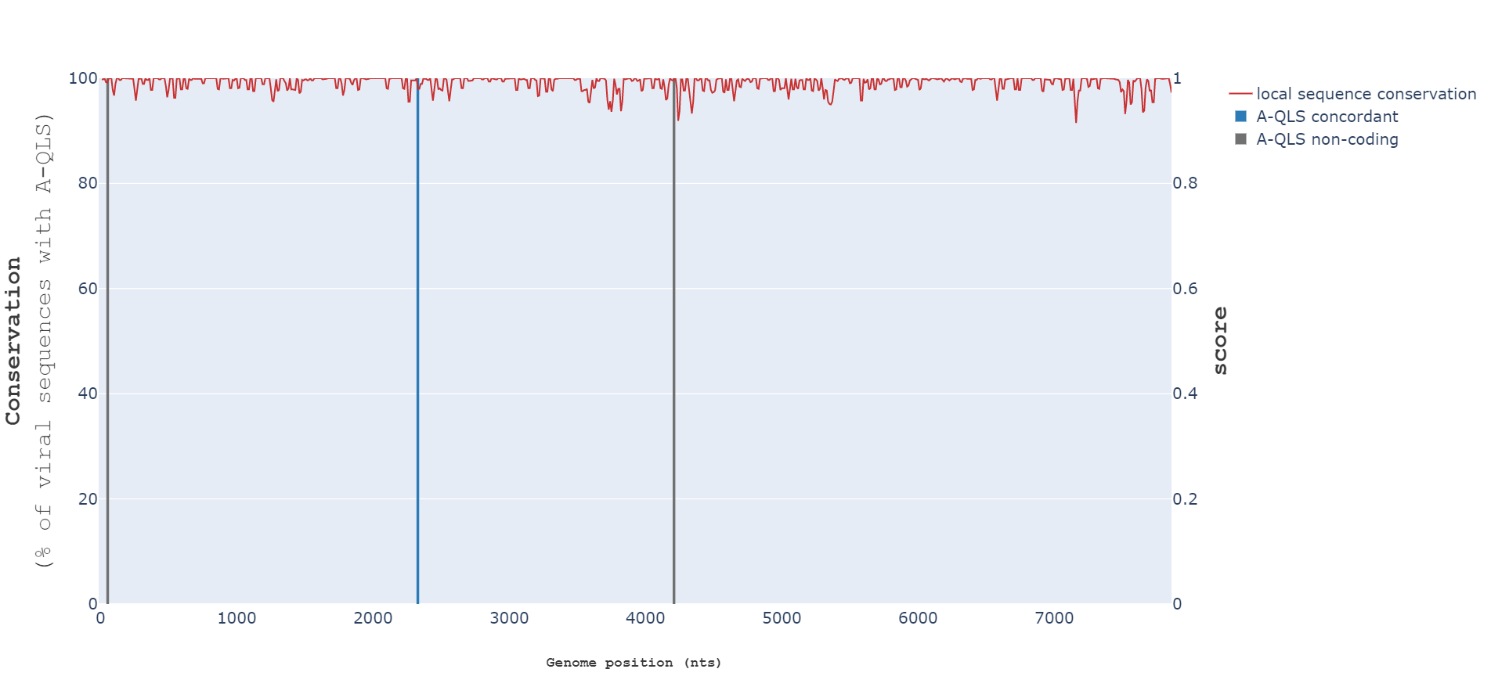


**Fig S6. Conservation of A-QLSs in the HPV18 genome.** Concordant, discordant, and non-coding A-QLSs are indicated as blue, orange, and grey vertical bars, respectively. Local sequence conservation (LSC) of viral genomes is shown as a red broken line. The x-axis indicates genome position, the y-axis the conservation %.


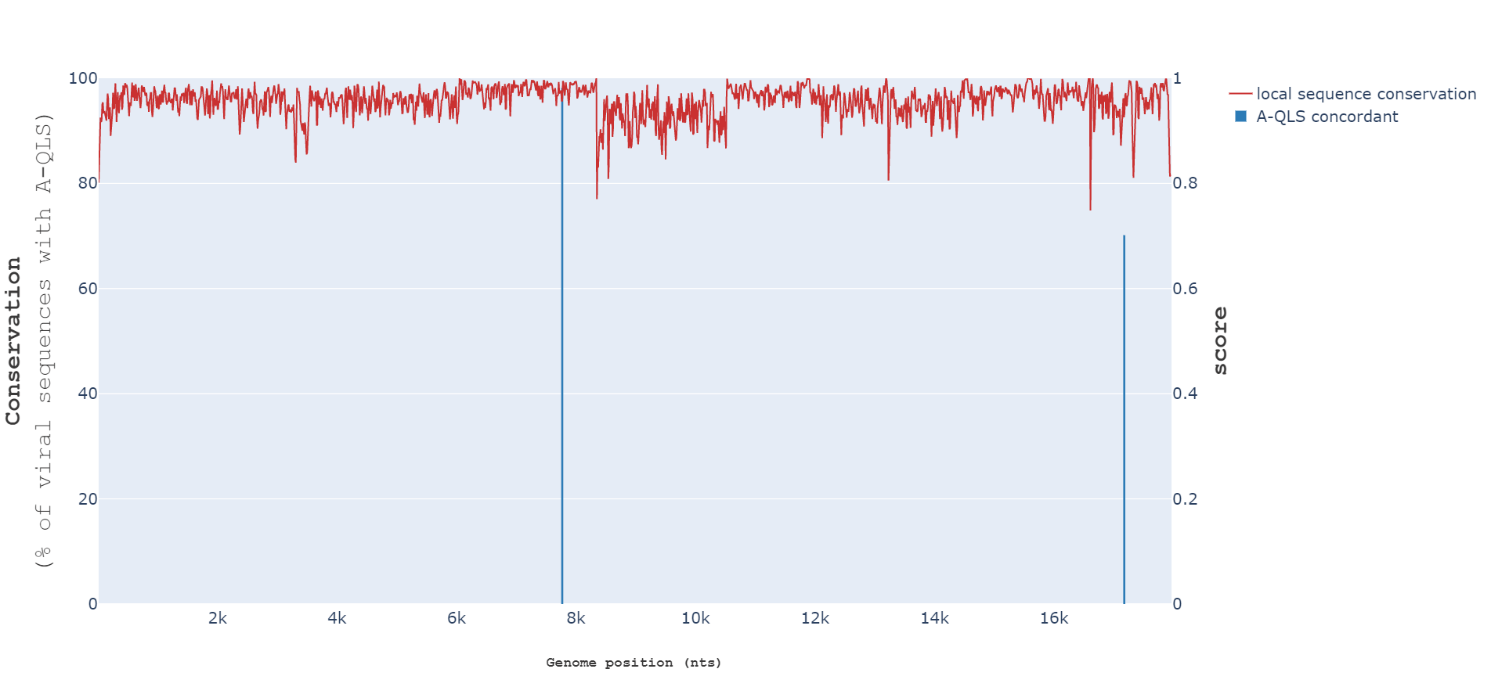


**Fig S7. Conservation of A-QLSs in the RotaC genome.** Concordant, discordant, and non-coding A-QLSs are indicated as blue, orange, and grey vertical bars, respectively. Local sequence conservation (LSC) of viral genomes is shown as a red broken line. The x-axis indicates genome position, the y-axis the conservation %.


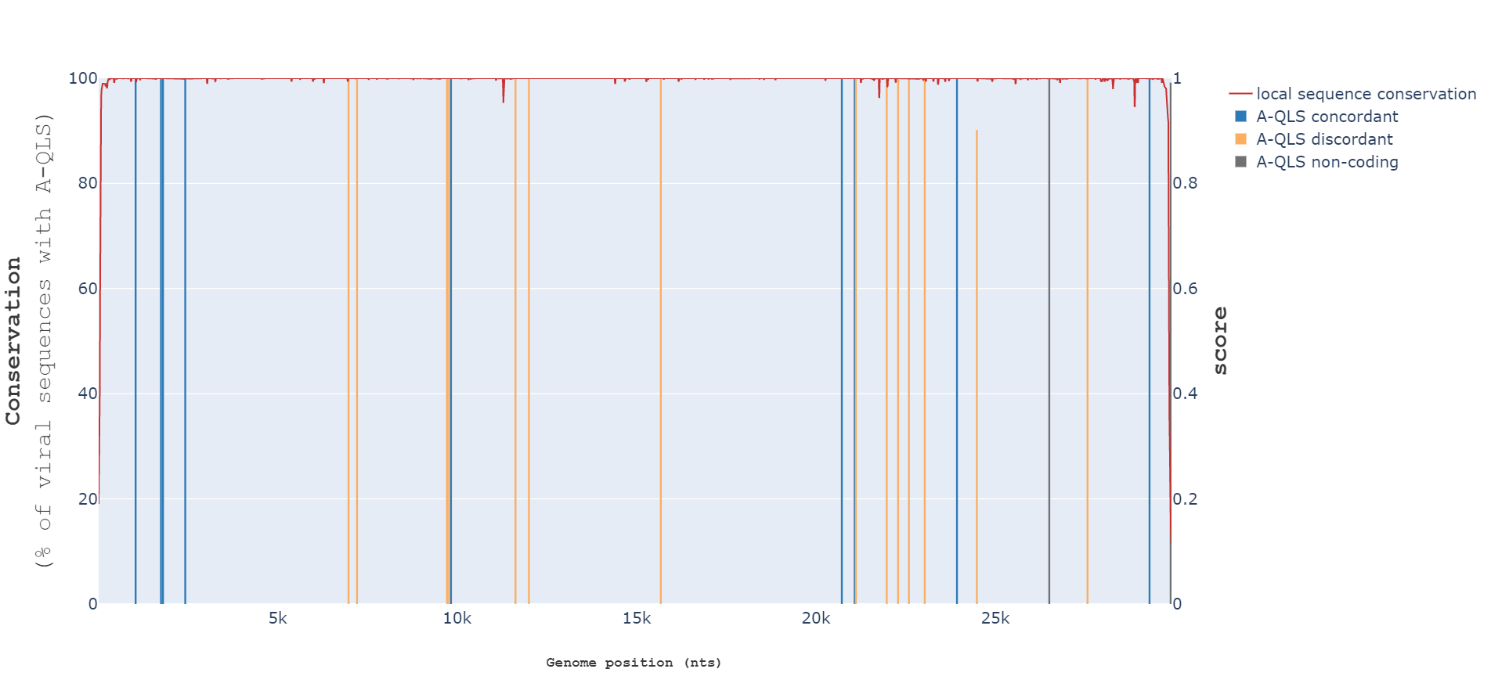


**Fig S8. Conservation of A-QLSs in the SARS-CoV-2 genome.** Concordant, discordant, and non-coding A-QLSs are indicated as blue, orange, and grey vertical bars, respectively. Local sequence conservation (LSC) of viral genomes is shown as a red broken line. The x-axis indicates genome position, the y-axis the conservation %.

**Table S4. Oligonucleotides used in this study.**

|  | Name | Sequence (5’-3’)*^a^* |
| --- | --- | --- |
| CD | **HHV6A-A** | AAAAACCTGAAAACATCGAAAAACCAAAAA |
|  | **HHV6A-T** | TTTTTGGTTTTTCGATGTTTTCAGGTTTTT |
|  | **HPV18a-A** | AAAAAAGGGAGTAACCGAAAACGGTCGGGACCGAAAACGGTGTATATAAAA |
|  | **HPV18a-T** | TTTTATATACACCGTTTTCGGTCCCGACCGTTTTCGGTTACTCCCTTTTTT |
|  | **HPV18b-A** | AAAATCATTTTTAAAAGGAACCCCCAAAAAAAA |
|  | **HPV18b-T** | TTTTTTTTGGGGGTTCCTTTTAAAAATGATTTT |
|  | **CTR** | TTGTCGTTAAAGTCTGACTGCGAGCTCTC |
| STOP ASSAYS | **HHV6A-A *Taq*** | AAAAACCTGAAAACATCGAAAAACCAAAAACTTCCCTGCATATAAGCAGCTGCTTTTTGCC |
|  | **HHV6A-T** | TTTTTGGTTTTTCGATGTTTTCAGGTTTTTAATAACTGCATATAAGCAGCTGCTTTTTGCC |
|  | **HPV18a-A** | AAAAAAGGGAGTAACCGAAAACGGTCGGGACCGAAAACGGTGTATATAAAAGATGTCTGCATATAAGCAGCTGCTTTTTGCC |
|  | **HPV18a-T** | ACATCTTTTATATACACCGTTTTCGGTCCCGACCGTTTTCGGTTACTCCCTTTTTTATATATGCATATAAGCAGCTGCTTTTTGCC |
|  | **HPV18b-A *Taq*** | AAAATCATTTTTAAAAGGAACCCCCAAAAAAAATTGTTCTGCATATAAGCAGCTGCTTTTTGCC |
|  | **HPV18b-T** | TTTTTTTTGGGGGTTCCTTTTAAAAATGATTTTAAGGCCTGCATATAAGCAGCTGCTTTTTGCC |
|  | **RotaC – A** | TGCTTATATGCAGGATCTGAGGGATAATTTTGTTTTGCTGCTTTTTTGAACATTTTAGTATTTCTAAATTTTTT*CCCTATAGTGAGTCGTATTA* |
|  | **RotaC – U** | TGCTTATATGCAGGATCTGAGGTTATGAAAAAATTTAGAAATACTAAAATGTTCAAAAAAGCAGCAAAACAAAA*CCCTATAGTGAGTCGTATTA* |
|  | **SARS-CoV-2a** | TGCTTATATGCAGGATCTGAGGGCACCTTTTTTAGCTTTTCCTTTTGTAACTTT*CCCTATAGTGAGTCGTATTA* |
|  | **SARS-CoV-2b** | TGCTTATATGCAGGATCTGAGGAAATCTTTAATTGGTGGTGTTTTGTAAATTTGTTT*CCCTATAGTGAGTCGTATTA* |
|  | **SARS-CoV-2c** | TGCTTATATGCAGGATCTGAGGTGATCTTTGAAATTTGGATCTTTGTCATCCAATTT*CCCTATAGTGAGTCGTATTA* |
|  | **Primer *Taq*** | CTGCATATAAGCAGCTGCTTTTTGCC |
|  | **CTR *Taq*** | TTGTCGTTAAAGTCTGACTGCGAGCTCTCAGATCCTGCATATAAGCAGCTGCTTTTTGCC |
|  | **RNA scrambled** | GUAACCGAUGAGUCUAUGCGAGCCCTCAGATCCTGCATATAAGCA |
|  | **Primer RT** | CCTCAGATCCTGCATATAAGCA |
|  | **Primer CTS** | TAGCAACAGACATACAAACTA |
|  | **HIV-1 CTS** | CTGGTCCTTTCCAAAGTGGATCTCTGCTGTCCCTGTAATAAACCCGAAAATTTTGAATTTTTGTAATTTGTTTTTGTAATTCTTTAGTTTGTATGACTGTTGCTA |

*^a^*The *Taq* and RT primer recognition sites are underlined; the T7 promoter site is reported in italic.
